# Supplementary material for: Magnetic Field Dynamic Strategies for the Improved Control of the Angiogenic Effect of Mesenchymal Stromal Cells
Source: Polymers (Basel). 2021 Jun 6;13(11):1883. doi: 10.3390/polym13111883 (PMC8201388; doi:10.3390/polym13111883)
Supplement: Supplementary file 1 [file polymers-13-01883-s001.zip › polymers-1171347-supplementary.pdf]

SUPPLEMENTARY INFORMATION

Magnetic Field Dynamic Strategies for the Improved Control of the Angiogenic Effect of Mesenchymal Stromal Cells

Ana C. Manjua [1,2], Joaquim M.S. Cabral [2,3], Frederico Castelo Ferreira [2,3,*] and Carla A.M. Portugal [1,*]

[1]LAQV-REQUIMTE, Department of Chemistry, NOVA School of Science and Technology, Universidade Nova de Lisboa, Campus da Caparica, 2829-516 Caparica, Portugal

[2]Department of Bioengineering and iBB—Institute for Bioengineering and Biosciences, Instituto Superior Técnico, Universidade de Lisboa, Av. Rovisco Pais, 1049-001 Lisboa, Portugal

[3]Associate Laboratory i4HB – Institute for Health and Bioeconomy, Instituto Superior Técnico, Universidade de Lisboa, Lisboa, Portugal

*Correspondence: frederico.ferreira@tecnico.ulisboa.pt (F.C.F.); cmp@fct.unl.pt (C.A.M.P.)

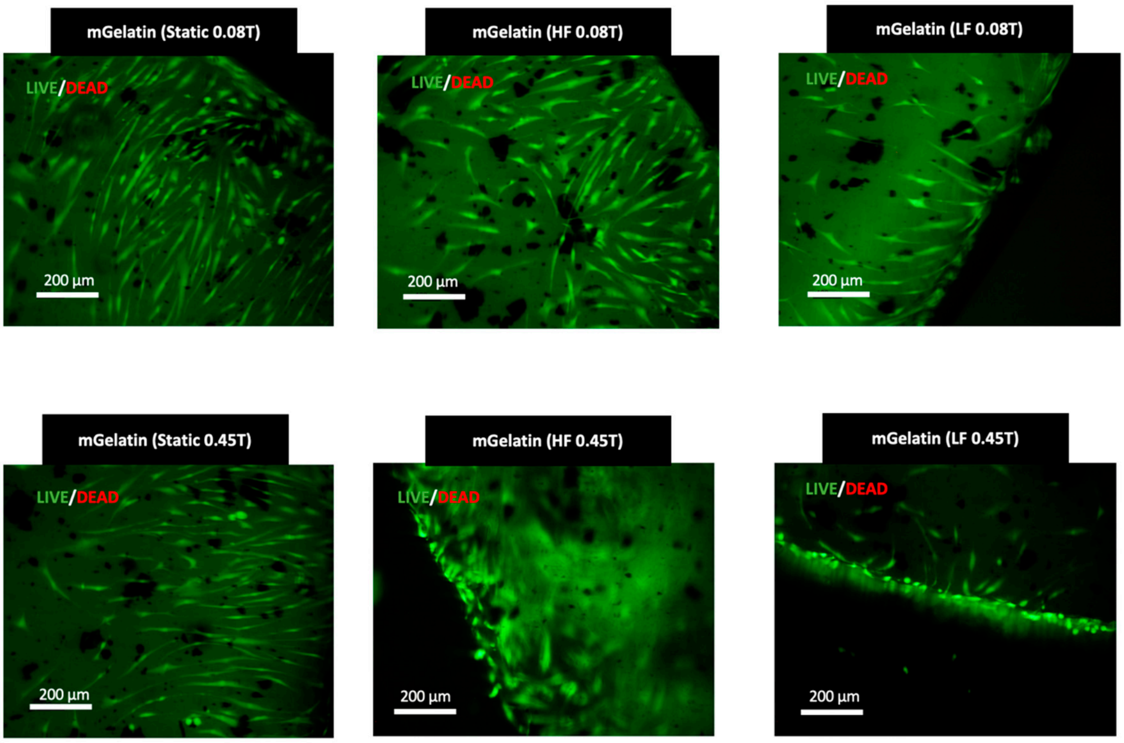

Figure S1. Representative images of MSC distribution in the magnetic gelatin scaffold under exposure to different magnetic field intensities (0.08 T, 0.45 T) and regimes (static, HF, LF). A LIVE/DEAD assay was performed and MSCs were stained with calcein (to identify living cells) and with ethidium bromide (to indicate dead cells). Scale bar: 200 µm. No significative cell patterning differences for MSCs exposed to static, LF or HF dynamic regimes were observed through comparative analysis of these images. However, a reduced MSCs density was observed upon exposure to LF conditions.

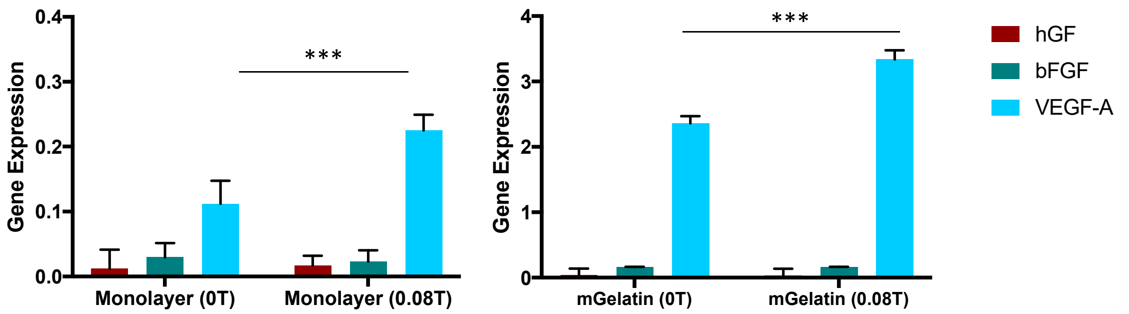

Figure S2. Magnetic field impact on the expression of the genes hGF, bFGF and VEGF-A by MSCs, cultured in monolayer (left side) and in the magnetic responsive gelatin scaffolds, mGelatin (right side). Magnetic exposure shows an impact on the expression of the gene VEGF-A but no effect is observed for the other angiogenic genes analyzed (bFGF, hGF). This analysis was performed under static magnetic field with intensity 0.08 T. An independent donor was also used in this analysis (Donor D: male, 42 years old, bone marrow sample isolated in 2015).

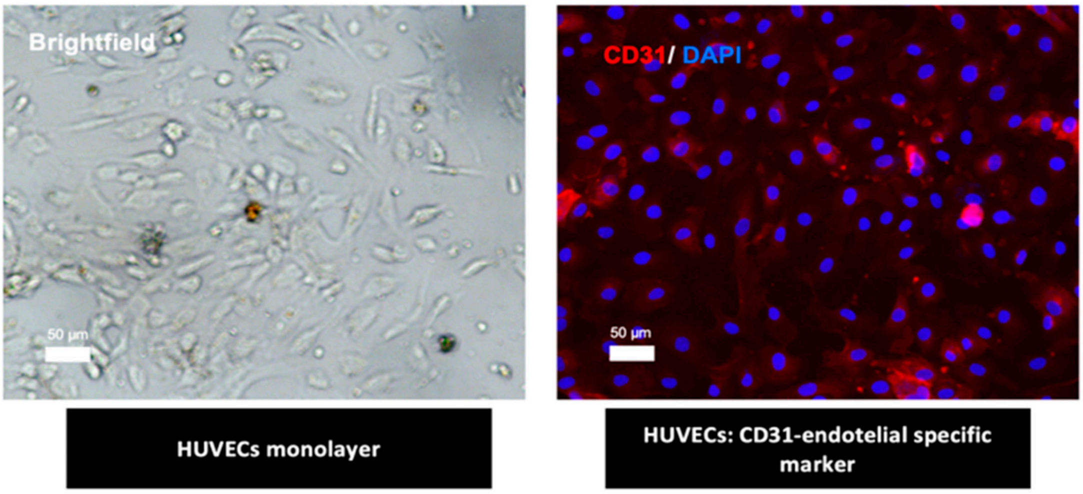

Figure S3. Representative images showing a randomly dispersed monolayer of HUVECs (brightfield image on the left, scale bar: 50 µm) and the identification of the endothelial lineage of the cells using CD31, specific marker of endothelial cells for immunofluorescence, and DAPI, to stain cell nuclei (immunofluorescence image on the right, scale bar: 50 µm).

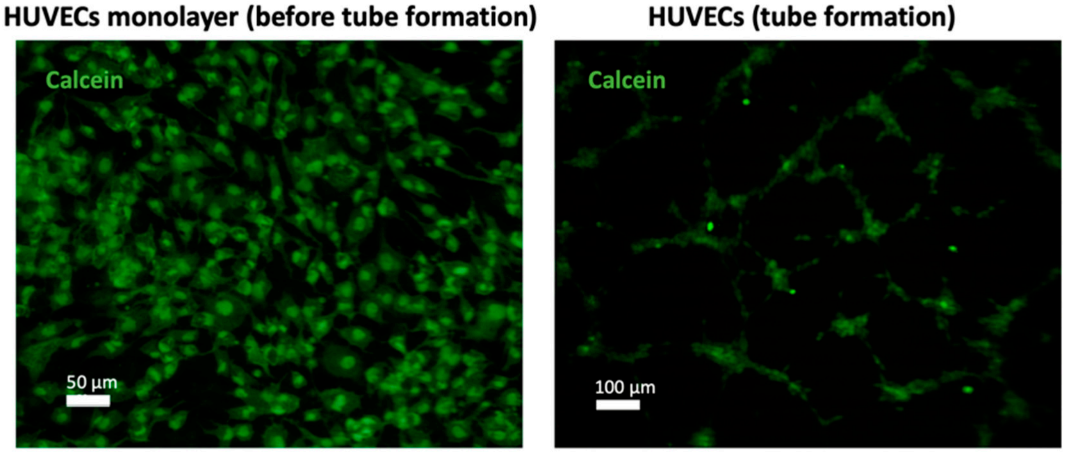

Figure S4. Representative images of HUVECs stained with calcein (indicator of living cells) to show morphological differences between HUVECs cultured in monolayer, before cell maturation in the tube formation assay with a random cell distribution (left side, scale bar: 50 µm) and during tube formation functional experiment (right side, scale bar: 100 µm), where tube-like structures are formed. During tube formation, HUVECs were supplemented with the conditioned media from MSCs exposed to magnetic stimulation.
